# Fabrication of ZnCo_2_O_4_-Zn(OH)_2_ Microspheres on Carbon Cloth for Photocatalytic Decomposition of Tetracycline

**DOI:** 10.3390/molecules29174054

**Published:** 2024-08-27

**Authors:** Sin-Ei Juang, Ning-Chien Chin, Yu-Cheng Chang, Chia-Man Chou

**Affiliations:** 1 Department of Materials Science and Engineering, Feng Chia University, Taichung 40724, Taiwan; juang5251@cgmh.org.tw (S.-E.J.); a111079@mail.tsmh.org.tw (N.-C.C.); 2 Department of Anesthesiology, Kaohsiung Chang Gung Memorial Hospital, Chang Gung University College of Medicine, Kaohsiung 83301, Taiwan; 3 Department of Orthopedics, Antai Tian-Sheng Memorial Hospital, Antai Medical Care Corporation, Pingtung 92842, Taiwan; 4 Department of Surgery, Taichung Veterans General Hospital, Taichung 40705, Taiwan; cmchou@vghtc.gov.tw; 5 College of Medicine, National Yang Ming Chiao Tung University, Taipei 11221, Taiwan; 6 Department of Post-Baccalaureate Medicine, National Chung Hsing University, Taichung 40227, Taiwan

**Keywords:** ZnCo_2_O_4_-Zn(OH)_2_ microspheres, carbon cloth, hydrothermal, thermal annealing, photocatalysts, methyl violet, tetracycline

## Abstract

Zinc cobalt oxide-zinc hydroxide (ZnCo_2_O_4_-Zn(OH)_2_) microspheres were successfully fabricated on carbon cloth via a sample hydrothermal method. The surface morphology of these microspheres and their efficacy in degrading methyl violet were further modulated by varying the thermal annealing temperatures. Adjusting the thermal annealing temperatures was crucial for controlling the porosity of the ZnCo₂O₄-Zn(OH)₂ microspheres, enhancing their photocatalytic performance. Various analytical techniques were utilized to evaluate the physical and chemical properties of the ZnCo_2_O_4_-Zn(OH)_2_ microspheres, including field-emission scanning electron microscopy, energy-dispersive spectroscopy, X-ray diffraction, field-emission transmission electron microscopy, X-ray photoelectron spectroscopy, and UV-vis spectroscopy. Compared to untreated ZnCo_2_O_4_-Zn(OH)_2_ microspheres, those subjected to thermal annealing exhibited increased specific surface area and light absorption capacity, rendering them highly effective photocatalysts under UVC light exposure. Subsequent studies have confirmed the superior performance of ZnCo_2_O_4_-Zn(OH)_2_ microspheres as a reusable photocatalyst for degrading methyl violet and tetracycline. Furthermore, trapping experiments during the photodegradation process using ZnCo₂O₄-Zn(OH)₂ microspheres identified hydroxyl radicals (·OH) and superoxide radicals (·O₂⁻) as the primary reactive species.

## 1. Introduction

Spinel-structured photocatalysts are gaining attention for their cost-effectiveness, durability, and strong photoelectrochemical response, making them prime for enhancing solar energy capture [1,2,3]. Various AB_2_O_4_ spinels, such as ZnFe_2_O_4_ and ZnCo_2_O_4_, have been explored for applications ranging from gas sensing to energy storage and degradation of pollutants under visible light [4,5,6]. ZnCo_2_O_4_ is classified as a p-type semiconductor due to its spinel crystal structure and is notable for its versatility, including roles in Li-ion batteries, catalysis, and supercapacitors [7,8]. ZnCo_2_O_4_-based sensors have shown exceptional sensitivity to various gases, likely due to their high surface area [9]. The morphology of the nanoparticles significantly impacts gas sensing performance, highlighting the importance of optimizing ZnCo₂O₄ sensors for enhanced sensitivity and lower operating temperatures [10,11]. Diverse synthesis methods like hydrothermal and microwave-assisted heating techniques have been developed, offering efficiency and cost-effectiveness for oxide material structures [12,13,14,15]. ZnCo_2_O_4_′s effective use in breaking down organic pollutants showcases its potential for environmental cleanup [16,17,18]. The evolution from bulk to porous structures, offering more active sites and better light absorption, marks a significant advancement [19]. The annealing-based self-sacrificial templating emerges as a cost-effective, high-performance method for fabricating these porous photocatalysts, emphasizing the importance of precursor selection in achieving superior product quality [16,20,21]. Previous studies have demonstrated the successful preparation of ZnCo_2_O_4_ nanostructures on carbon cloth for electrodes for supercapacitors and lithium-ion batteries [22,23,24]. However, their applications on carbon cloth for photocatalytic degradation remain infrequent.

Carbon cloth, a carbon filament textile, offers high conductivity, mechanical strength, and flexibility, making it ideal for flexible energy storage systems [25,26,27]. Despite a low surface area and few electroactive sites, carbon cloth is a flexible substrate for electrode materials [28,29,30]. Notably, Wang et al.’s oxidative method with hydrogen peroxide and sulfuric acid introduces oxygen-containing groups onto carbon cloth, enhancing its function [31]. Kordek et al., Liu et al., and Zhao et al. employed various complex activation methods to enhance the oxygen electrocatalytic activities of carbon cloth, each achieving improved performance through surface modification techniques, such as etching, calcining, plasma treatment, and doping with heteroatoms [32,33]. Therefore, surface modification technology can further improve carbon cloth’s specific surface area and active sites, improving its application in various fields. Past research has shown that combining carbon cloth and ZnCo_2_O_4_ nanostructures creates a high-capacity, flexible anode with excellent cycle stability and rate performance, thus forming a highly flexible lithium-ion battery with excellent electrochemical properties [23,34]. In this study, combining carbon cloth and ZnCo_2_O_4_-Zn(OH)_2_ microspheres is expected to be further used in the photocatalytic degradation of methyl violet and tetracycline, simplifying the subsequent recycling process.

ZnCo₂O₄-Zn(OH)₂ microspheres fabricated on carbon cloth through a simplified hydrothermal process demonstrated enhanced photocatalytic degradation of pollutants such as methyl violet, attributed to optimized porosity achieved by varying thermal annealing temperatures. Comprehensive characterization showed that annealed microspheres possess superior surface area, enhanced light absorption, and improved charge carrier separation, making them highly effective under UVC light. These microspheres demonstrated efficiency as reusable photocatalysts, with superoxide and hydroxyl radicals identified as the primary reactive species in pollutant degradation.

## 2. Results and Discussion

**Figure 1:**
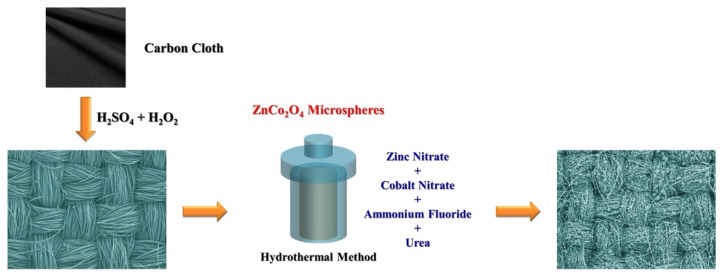
Describe the reaction mechanism and how to prepare ZnCo_2_O_4_-Zn(OH)_2_ microspheres with the ratio of Zn/Co 1:2 on the carbon cloth.

Figure 1 is a detailed schematic diagram illustrating the growing ZnCo_2_O_4_-Zn(OH)_2_ microspheres on a carbon cloth substrate. Initially, carbon cloth undergoes a meticulous etching process using a mixture of sulfuric acid and hydrogen peroxide. This critical step creates a series of micropores on the surface, enhancing its texture and providing anchoring points that facilitate the subsequent nucleation and growth of ZnCo_2_O_4_-Zn(OH)_2_ microspheres. Following surface treatment, the carbon cloth underwent hydrothermal processing, forming and adhesion of ZnCo_2_O_4_-Zn(OH)_2_ microspheres on the etched surface. The reaction process was performed at a controlled temperature of 120 °C and maintained for 2 h, ensuring uniform growth of microspheres. This meticulously designed process successfully integrated ZnCo_2_O_4_-Zn(OH)_2_ microspheres onto carbon cloth, enhancing surface properties and leveraging the synergistic effects between the carbon cloth and the microspheres, thus laying the foundation for potential applications in photocatalytic degradation.

**Figure 2:**
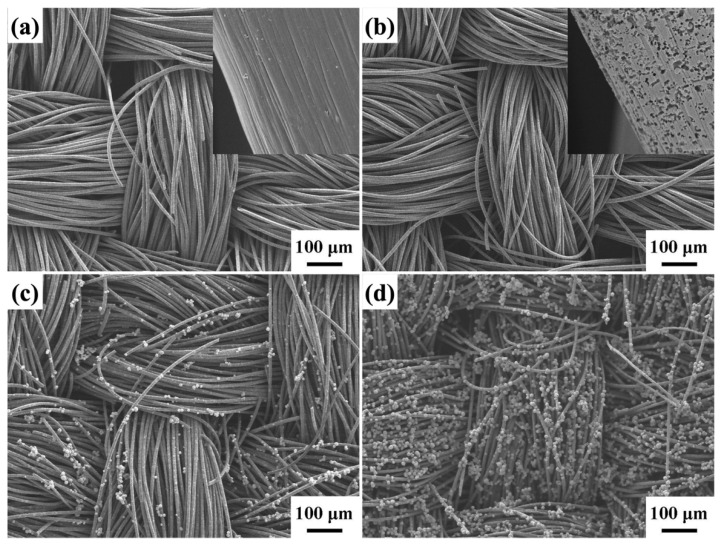
The FESEM images of (**a**) carbon cloth (without etching), (**b**) carbon cloth (etching), and ZnCo_2_O_4_-Zn(OH)_2_ microspheres grown on the carbon cloth (**c**) without etching and (**d**) etching.

Figure 2a,b shows FESEM images of carbon cloth before and after soaking in a solution containing hydrogen peroxide and sulfuric acid. Before shaking, the carbon cloth surface is quite smooth. However, after soaking, the surface of the carbon cloth displays holes of various sizes. Subsequently, the two types of carbon cloth are placed in a ZnCo_2_O_4_-Zn(OH)_2_ reaction precursor at a reaction temperature of 120 °C for 2 h by a facile hydrothermal process, as shown in Figure 2c,d. These images reveal that after soaking in the sulfuric acid and hydrogen peroxide mixture, the ZnCo_2_O_4_-Zn(OH)_2_ microspheres on the surface of the carbon cloth exhibit a higher density. This result proves that soaking the carbon cloth in a solution containing hydrogen peroxide and sulfuric acid creates more pores on its surface and aids in the subsequent growth of ZnCo_2_O_4_-Zn(OH)_2_ microspheres.

**Figure 3:**
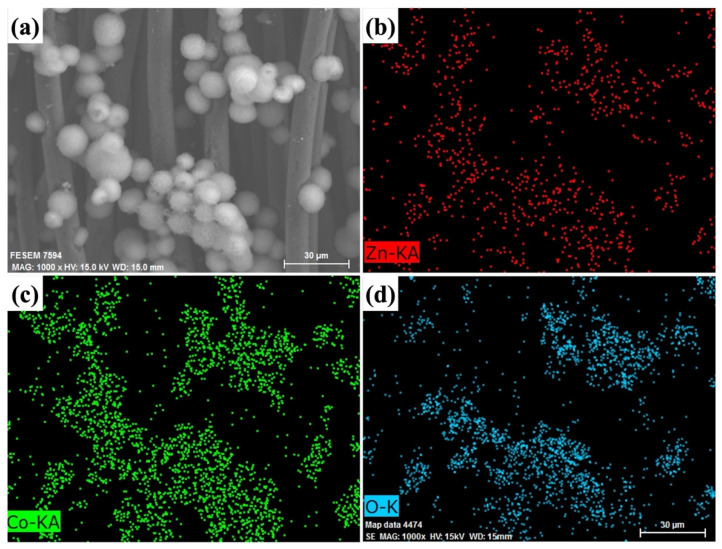
The (**a**) FESEM and (**b**–**d**) FESEM-EDS mapping images of ZnCo_2_O_4_-Zn(OH)_2_ microspheres grown on the carbon cloth (etching).

Additionally, high-resolution FESEM images (as seen in Figure 3a) depict the presence of ZnCo_2_O_4_-Zn(OH)_2_ microspheres. The distribution of specific elements within these microspheres is further elucidated via FESEM-EDS elemental mapping, as shown in Figure 3b–d. Examination of these images reveals that the microspheres are composed of zinc (Zn), cobalt (Co), and oxygen (O), with these components being uniformly distributed throughout. Consequently, this highlights the specific elemental composition of the ZnCo_2_O_4_-Zn(OH)_2_ microspheres.

**Figure 4:**
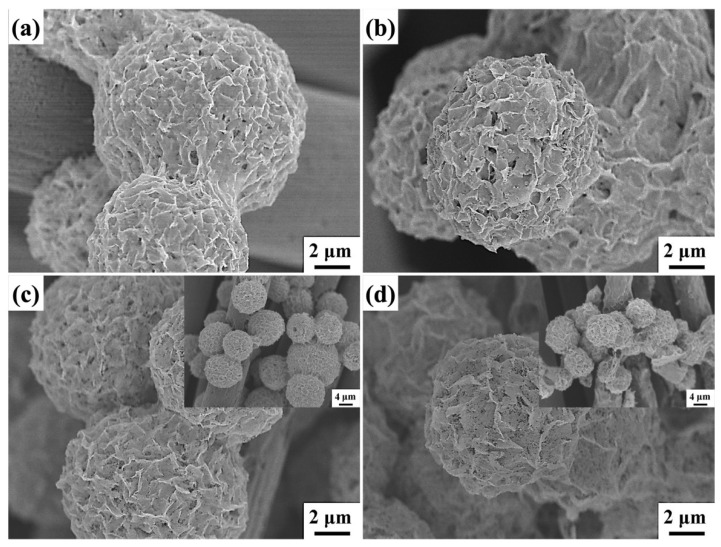
The FESEM images of ZnCo_2_O_4_-Zn(OH)_2_ microspheres grown on the carbon cloth under the different annealing temperatures. The annealing temperatures are (**a**) without, (**b**) 450 °C, (**c**) 550 °C, and (**d**) 650 °C, respectively.

Figure 4 shows FESEM images of ZnCo_2_O_4_-Zn(OH)_2_ microspheres subjected to different thermal annealing temperatures for 2 h. The thermal annealing temperatures are (a) without, (b) 450 °C, (c) 550 °C, and (d) 650 °C, respectively. As the annealing temperature rises, there is a progressive emergence of porosity on the surface. The surface morphology of the ZnCo_2_O_4_-Zn(OH)_2_ microspheres remains unchanged compared to the unannealed samples, with the only noticeable difference being the appearance of porosity at an annealing temperature of 550 °C for 2 h. When the annealing temperature rises to 650 °C, it becomes apparent that some microspheres have collapsed. This occurrence drastically decreases the reactive surface area, negatively impacting the efficiency of subsequent photocatalytic reactions. The structural collapse observed at higher temperatures underscores the material’s thermal instability, highlighting the necessity for meticulous optimization of the thermal annealing process to preserve the desired functional properties of the microspheres. Hence, the BET analyzer can assess the specific surface area of ZnCo_2_O_4_-Zn(OH)_2_ microspheres before and after thermal annealing at 550 °C for 2 h. The surface area of the microspheres was measured at 20.78 m^2^ g^−1^ before thermal annealing, and following the annealing process, this value rose to 31.29 m^2^ g^−1^. This result indicates that thermal annealing effectively enhances the specific surface area of the microspheres.

**Figure 5:**
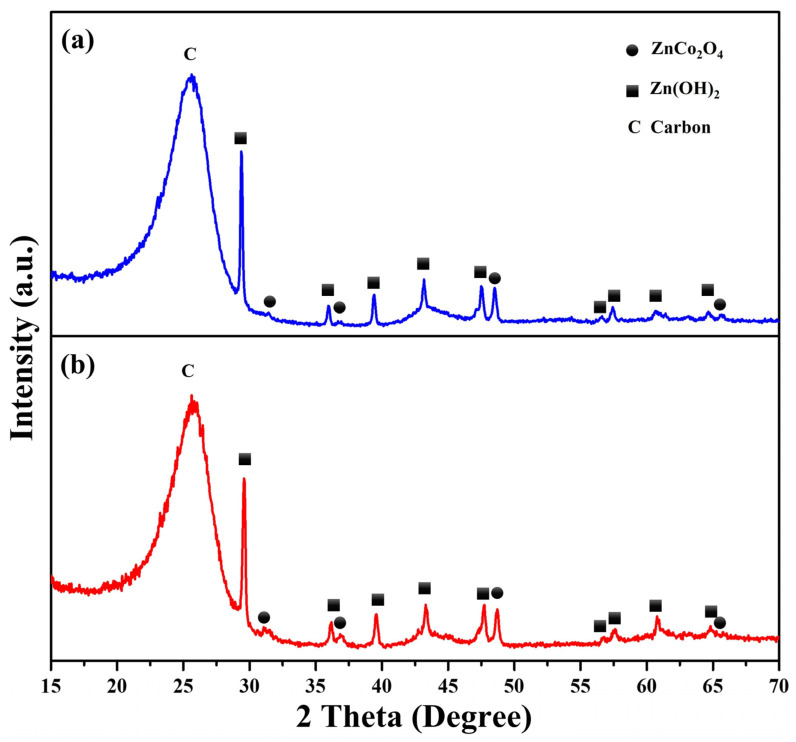
The XRD patterns of ZnCo_2_O_4_-Zn(OH)_2_ microspheres grown on the carbon cloth (**a**) without thermal annealing and (**b**) thermal annealing at 550 °C for 2 h, respectively.

X-ray diffraction (XRD) was employed for the examination of the crystalline structure of the ZnCo_2_O_4_-Zn(OH)_2_ microspheres (without thermal annealing) and ZnCo_2_O_4_-Zn(OH)_2_ microspheres (thermal annealing at 550 °C for 2 h), as shown in Figure 5. The broad diffraction peak observed for the carbon cloth is likely due to the presence of a graphitized carbon peak. The diffraction angles of 29.6°, 35.5°, 43.3°, 47.5°, 57.2°, 58.0°, 60.9°, and 64.5° can be observed in Zn(OH)_2_, corresponding to the (031), (211), (350), (181), (0120), (2111), (510), (152), and (1141) planes of orthorhombic Zn(OH)_2_ (PDF No. 00-020-1437), respectively. The diffraction angles of 31.2°, 36.8°, 48.9°, and 65.2° can be observed in ZnCo_2_O_4_, corresponding to the (220), (311), (331), and (440) planes of cubic ZnCo_2_O_4_ (PDF No. 00-023-1390), respectively. It is evident that the peak intensity of ZnCo_2_O_4_ in ZnCo_2_O_4_-Zn(OH)_2_ microspheres is weakened, indicating that the content of ZnCo_2_O_4_ is lower and the crystallinity is not as strong as that of Zn(OH)_2_ when Zn(OH)_2_ and ZnCo_2_O_4_ are combined. This result confirms the successful generation of ZnCo_2_O_4_-Zn(OH)_2_ microspheres.

**Figure 6:**
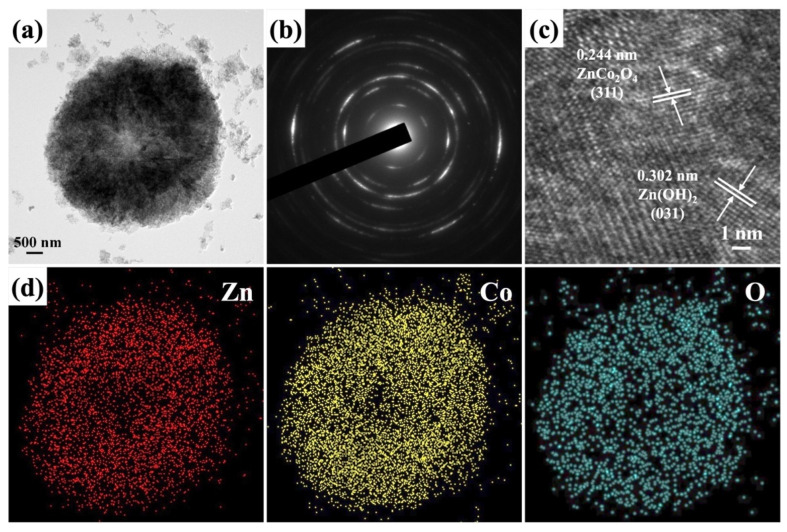
The (**a**) FETEM image, (**b**) SAED pattern, (**c**) HRTEM image, (**d**) EDS-mapping images of ZnCo_2_O_4_-Zn(OH)_2_ microspheres grown on the carbon cloth under the annealing temperature of 550 °C.

The FETEM image in Figure 6a reveals a microspherical structure of ZnCo_2_O_4_-Zn(OH)_2_, which aligns with the SEM results. This configuration is characterized by the arrangement of numerous sheets stacked on each other. The typical SAED pattern (Figure 6b) further confirms the polycrystalline nature of the ZnCo_2_O_4_-Zn(OH)_2_ microsphere. The major diffraction ring closely matches the orthorhombic Zn(OH)_2_ (PDF No. 00-020-1437) and cubic ZnCo_2_O_4_ (PDF No. 00-023-1390) crystal structures. The HRTEM image of the ZnCo_2_O_4_-Zn(OH)_2_ microsphere (Figure 6c) displays crystal lattice fringes characterized by two discernible interplanar spacings: 0.302 nm and 0.244 nm. These can be attributed to the (031) crystallographic plane of the orthorhombic phase of Zn(OH)_2_ and the (311) crystallographic plane of the cubic phase of ZnCo_2_O_4_. Figure 6d reveals the corresponding elemental mapping images of the ZnCo_2_O_4_-Zn(OH)_2_ microsphere, showing the distribution of Zn, Co, and O elements. This result indicates that Zn, Co, and O define the ZnCo_2_O_4_-Zn(OH)_2_ microsphere composition.

**Figure 7:**
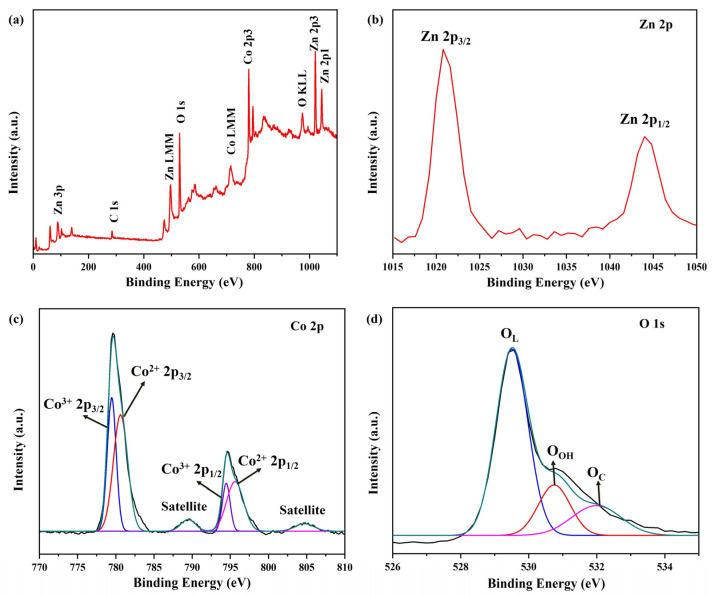
XPS (**a**) survey, (**b**) Zn 2p, (**c**) Co 2p, and (**d**) O 1s spectra of ZnCo_2_O_4_-Zn(OH)_2_ microspheres under the annealing temperature of 550 °C.

To analyze the elemental composition and valence state distribution on the surface of ZnCo_2_O_4_-Zn(OH)_2_ microspheres, X-ray photoelectron spectroscopy (XPS) was employed. Figure 7a displays the full range XPS spectrum of ZnCo_2_O_4_-Zn(OH)_2_ microspheres annealed at 550 °C, exhibiting distinct peaks corresponding to C, Zn, Co, and O. These peaks are consistent with the TEM-EDS observations, further confirming the presence of these elements. The carbon element is believed to have its source in the pump oil present in the vacuum system of the XPS equipment, carbon cloth, or an organic layer that has been applied to the surface of the sample. The high-resolution XPS spectrum (Figure 7b) of Zn 2p, showing peaks at 1020.9 eV and 1044.1 eV for Zn 2p_3/2_ and Zn 2p_1/2,_ respectively, confirms the presence of Zn^2+^ in the ZnCo_2_O_4_-Zn(OH)_2_ structure [35,36]. Figure 7c reveals the high-resolution XPS spectrum of Co 2p of ZnCo_2_O_4_, which can be deconvolved into four different states: Co^3+^ at 779.5 eV (2p_3/2_) and 794.5 eV (2p_1/2_), and Co^2+^ at 780.6 eV (2p_3/2_) and 795.7 eV (2p_1/2_). Additionally, two vibrational satellite peaks for Co^2+^ are located at 789.6 eV near the Co 2p_3/2_ band and 804.8 eV near the Co 2p_1/2_ band, consistent with previous literature [37,38]. The O 1 s spectrum of the synthesized ZnCo_2_O_4_-Zn(OH)_2_ microspheres (Figure 7d) reveals a primary peak at 529.5 eV corresponding to lattice oxygen (O_L_), along with shoulder peaks at 530.8 eV and 532.1 eV attributed to surface hydroxyl groups (O_OH_) and chemisorbed oxygen (O_C_) [37,38].

**Figure 8:**
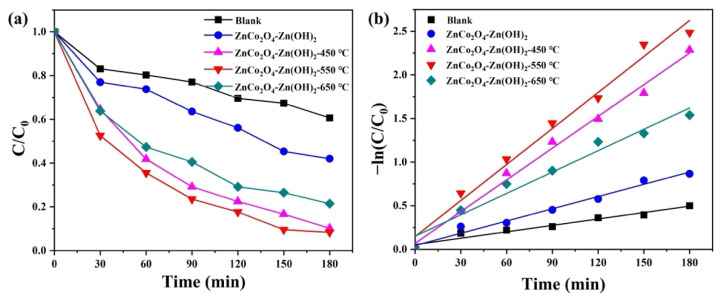
(**a**) Photocatalytic efficiency and (**b**) kinetic plot of as-prepared photocatalysts for MV solution under the UVC light irradiation.

To comprehend the correlation between the photocatalytic efficiency of ZnCo_2_O_4_-Zn(OH)_2_ microspheres under different annealing temperatures, the photocatalytic activity in degrading methyl violet (MV), an organic pollutant commonly found in the textile industry was evaluated [39]. ZnCo_2_O_4_-Zn(OH)_2_ microspheres were grown on a 2.5 cm × 1.5 cm carbon cloth substrate as photocatalytic samples. The photocatalytic efficiency of ZnCo_2_O_4_-Zn(OH)_2_ microspheres at different annealing temperatures was evaluated by the degradation of MV by UVC light (253 nm, 10 W), as shown in Figure 8a. The time variation of MV concentration was monitored by examining the change in maximum absorbance at 587 nm in UV-vis spectroscopy. The photodegradation percentages of MV were 58.0% (without annealing), 89.8% (450 °C), 91.7% (550 °C), and 78.6% (650 °C). When the annealing temperature was below 550 °C, a decrease in maximum absorbance was observed with increasing irradiation time and annealing temperature. ZnCo_2_O_4_-Zn(OH)_2_ microspheres (550 °C) exhibited the highest photocatalytic activity in MV decomposition. The photocatalytic degradation process conformed to pseudo-first-order kinetics, and the plot of −ln(C/C_0_) versus irradiation time (t) showed a pseudo-first-order linear relationship (Figure 8b), where C_0_ is the initial concentration of MV and C is the actual concentration of MV at time t. The slope of the pseudo-first-order linear line is the apparent rate constant (k, min^–1^) of the photocatalytic reaction. The rate constants of ZnCo_2_O_4_-Zn(OH)_2_ microspheres at different annealing temperatures were calculated to be 0.03298 (without annealing), 0.05002 (450 °C), 0.08519 (550 °C), and 0.07241 min^–1^ (650 °C), respectively. ZnCo_2_O_4_-Zn(OH)_2_ microspheres (550 °C) displayed the highest photocatalytic efficiency in MV photodegradation under UVC light irradiation. The rate constant (k) of ZnCo_2_O_4_-Zn(OH)_2_ microspheres (550 °C) was about 2.58 times higher than that of the non-annealed ones. This phenomenon is attributed to the formation of porosity on the surface of ZnCo_2_O_4_-Zn(OH)_2_ microspheres after annealing, which increases the active sites. However, when the annealing temperature is too high, it can cause the structure to collapse, leading to a decrease in active sites, which is consistent with the observations from SEM results.

**Figure 9:**
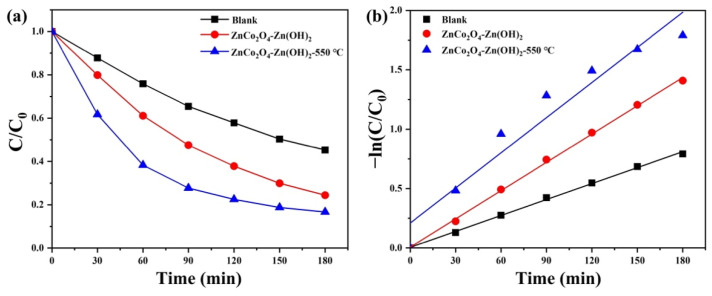
(**a**) Photocatalytic efficiency and (**b**) kinetic plot of the photocatalysts in treating TC solution under UVC light irradiation.

We chose tetracycline (TC) as an antibiotic to illustrate that ZnCo_2_O_4_-Zn(OH)_2_ microspheres can also be used for photocatalytic antibiotic degradation. Tetracycline (TC), a widely used antibiotic effective against various infections, is prevalent in water bodies due to its use as a growth promoter in aquaculture and insufficient removal by traditional wastewater treatments [40,41]. As demonstrated in Figure 9a, we observe the degradation rates of ZnCo_2_O_4_-Zn(OH)_2_ microspheres before and after annealing when subjected to UVC light. The findings indicated that the microspheres that did not undergo the annealing process had a degradation rate of 75.6%. In contrast, those annealed at a temperature of 550 °C exhibited a rate of 83.3%. Figure 9b represents the pseudo-first-order linear relationship of the ZnCo_2_O_4_-Zn(OH)_2_ microspheres in the non- and annealed process. The reaction constants, corresponding to TC degradation over the non-annealed microspheres and those annealed at 550 °C, were determined to be 0.00794 and 0.00986 min^−1^, respectively. Notably, the microspheres that underwent annealing at 550 °C showed superior photocatalytic activity, with their reaction constant being 1.24 times greater than their non-annealed counterparts when exposed to UVC light. This suggests an enhancement in photocatalytic efficiency due to the annealing process.

**Figure 10:**
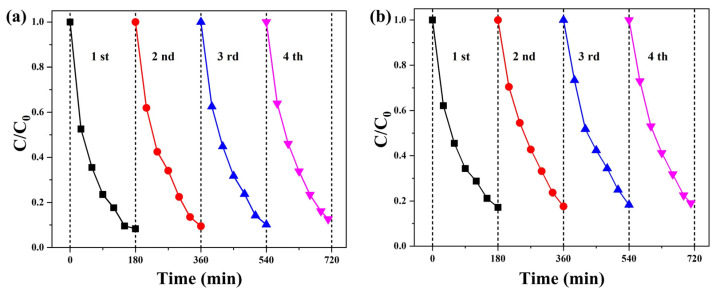
Recycle experiments of ZnCo_2_O_4_-Zn(OH)_2_ microspheres for (**a**) MV and (**b**) TC solution under UVC light irradiation.

**Figure 11:**
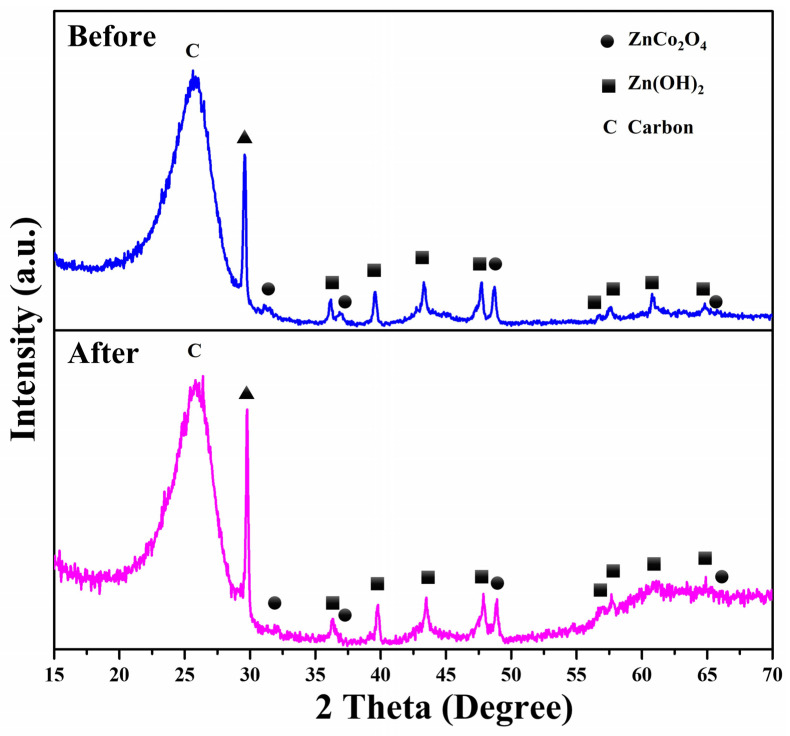
XRD patterns of ZnCo_2_O_4_-Zn(OH)_2_ microspheres (550 °C) before (blue line) and after (purple line) four cycles of TC degradation.

The recyclability of ZnCo_2_O_4_-Zn(OH)_2_ microspheres (550 °C) was examined through repeated experiments involving the degradation of MV and TC solutions under UVC light irradiation, as shown in Figure 10. In the case of the MV solution (Figure 10a), the photocatalytic efficiency remained consistently high across four cycles, with efficiencies of 91.7%, 90.5%, 89.9%, and 87.4%, respectively. Similarly, the photocatalytic efficiency maintained a steady rate for the TC solution (Figure 10b), with 82.8%, 82.4%, 81.7%, and 80.9% across the four cycles. Even after four cycles of use, the decline in the photocatalytic efficiency of the ZnCo_2_O_4_-Zn(OH)_2_microspheres was insignificant, demonstrating their durability and consistent performance. This result suggests that the ZnCo_2_O_4_-Zn(OH)_2_ microspheres, heated at 550 °C, possess a long lifespan as photocatalysts, maintaining high activity and reusability. The ZnCo_2_O_4_-Zn(OH)_2_ microspheres were also directly cultivated on a carbon cloth. In the meantime, the tested ZnCo_2_O_4_-Zn(OH)_2_ microspheres (Figure 11) displayed remarkable consistency in the XRD patterns before and after degradation tests, validating their high resistance to photo-corrosion and stability. This result suggests that ZnCo_2_O_4_-Zn(OH)_2_ microspheres have the promising potential for repeated use in practical applications, a highly desirable characteristic for sustainable and efficient photocatalysts. This resilience ensures their longevity and enhances their cost-effectiveness, making them a compelling choice for environmental applications. This unique growth method simplifies recycling and provides a stable and economical photocatalyst platform.

**Figure 12:**
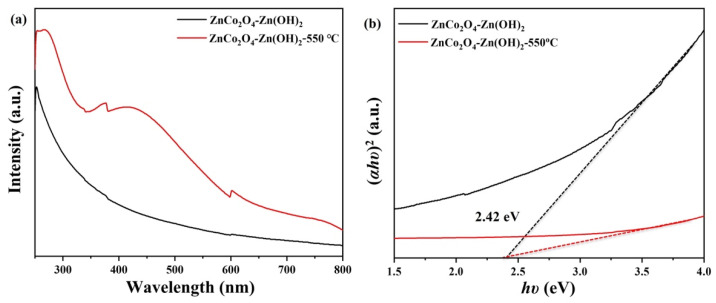
(**a**) UV-vis spectra and (**b**) Tauc plot of ZnCo_2_O_4_-Zn(OH)_2_ microspheres and ZnCo_2_O_4_-Zn(OH)_2_ microspheres (550 °C).

The optical properties of the ZnCo_2_O_4_-Zn(OH)_2_ microspheres, both with and without thermal annealing, were analyzed using UV–visible spectroscopy. As shown in Figure 12a, the ZnCo_2_O_4_-Zn(OH)_2_ microspheres that underwent thermal annealing at 550 °C demonstrated a significantly superior light absorption capacity within the spectral range spanning from 250 to 800 nm compared to those that did not undergo thermal annealing. The improved light absorption spectrum observed in the annealed microspheres offers advantages for maximizing solar energy utilization, thereby enhancing the photocatalytic degradation process. The energy band gaps (E_g_) were established utilizing the Tauc relationship, represented by the equation: αhν = A (E_g_ − hν)^1/n^ [42,43]. In the provided equation, A, α, ν, E_g_, and h represent constants: the constant, the absorption coefficient, the frequency of light, the band gap energy, and Planck’s constant, respectively. The variable “n” denotes a property of the semiconductor material, taking a value of 2 for indirect bandgap semiconductors and 1/2 for direct bandgap semiconductors, as illustrated in Figure 12b. The energy band gap value of the ZnCo_2_O_4_-Zn(OH)_2_ microspheres, both with and without annealing, was computed to be approximately 2.42 eV. These data validate that the energy gap remains relatively constant throughout the thermal annealing process. Moreover, a straightforward thermal annealing technique can offer a high specific surface area and a more extensive optical absorption spectrum at a suitable temperature. This method significantly enhances the photocatalytic degradation of MV or TC solutions, improving their overall performance and effectiveness.

**Figure 13:**
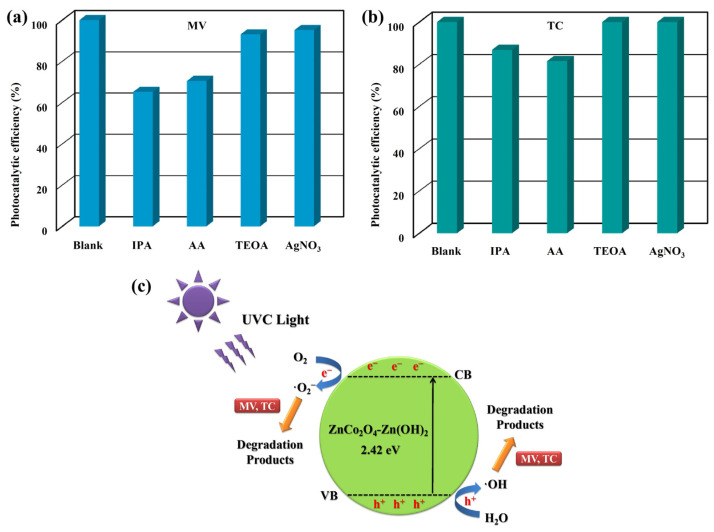
Photocatalytic activities of ZnCo_2_O_4_-Zn(OH)_2_ microspheres (550 °C) for (**a**) MV (blue bar) and (**b**) TC (teal bar) solutions with four scavengers under UVC light irradiation. (**c**) Describe in detail the reaction mechanism for photocatalytic degradation of MV and TC using ZnCo_2_O_4_-Zn(OH)_2_ microspheres.

Four radical scavengers were introduced into the photocatalytic reaction to investigate the underlying mechanism of the photocatalysts of ZnCo_2_O_4_-Zn(OH)_2_ microspheres during the photodegradation of MV or TC solution, as shown in Figure 13a,b. Isopropyl alcohol (IPA), L-ascorbic acid (AA), triethanolamine (TEOA), and silver nitrate (AgNO_3_) were utilized as scavengers to impede hydroxyl radicals (·OH), superoxide radical anions (·O^2−^), holes (h^+^), and electrons (e^–^), respectively [44,45,46,47]. Adding IPA and AA scavengers to the photocatalytic reaction leads to a notable reduction in the photocatalytic efficiency. This outcome provides evidence that hydroxyl radicals (·OH) and superoxide radicals (·O^2−^) are the primary active species involved in the photodegradation of TC. Potential reactions that may occur during the photocatalytic degradation of MV or TC solutions over ZnCo_2_O_4_-Zn(OH)_2_ microspheres can be outlined as a schematic diagram, as shown in Figure 13c. When the ZnCo_2_O_4_-Zn(OH)_2_ microspheres are exposed to UVC light with photon energy (hv) exceeding their band gap, an electron (e^–^) in the valence band (VB) can be promoted to the conduction band (CB), creating a hole in the VB and generating electron-hole pairs. Photogenerated electrons can interact with oxygen molecules on the surface, forming superoxide radical anions (·O_2_^–^). These can then react with water molecules absorbed on the surface, producing hydroxyl radicals (·OH). Moreover, the photogenerated holes may combine with H_2_O molecules, causing their dissociation into··OH radicals. These superoxide radical anions and hydroxyl radicals are recognized as potent oxidants responsible for the decomposition of MV or TC molecules.

## 3. Materials and Methods

### 3.1. Materials and Chemicals

Carbon cloth was procured from As One International (Santa Clara, CA, USA) as a commercial source. The chemicals were sourced from commercial suppliers and were used without requiring additional purification. Sulfuric acid (H_2_SO_4_, 95–97%), hydrogen peroxide (H_2_O_2_, 35%), isopropanol (C_3_H_8_O, 95%), and ethanol (C_2_H_5_OH, 99.5%) were acquired from Echo Chemical (Miaoli County, Taiwan). Zinc nitrate hexahydrate (Zn(NO_3_)_2_·6H_2_O, 98%), cobalt(II) nitrate hexahydrate (Co(NO_3_)_2_·6H_2_O, 98%), ammonium fluoride (NH_4_F, 98%), methyl violet (C_24_H_28_N_3_Cl, 99.5%), urea (CON_2_H_4_, 99%), triethanolamine (TEOA, C_6_H_15_NO_3_, 99+%), and L-ascorbic acid (C_6_H_8_O_6_, 99%) were acquired from Sigma-Aldrich (Darmstadt, Germany). Tetracycline (250 mg) was acquired from TWi Pharmaceuticals (Taipei City, Taiwan). Deionized (DI) water with a resistivity exceeding 18.3 MΩ was used to prepare all reaction solutions.

### 3.2. Fabrication of ZnCo_2_O_4_-Zn(OH)_2_ Microspheres

The carbon cloth was cut into (2.5 cm × 1.5 cm) sizes, soaked in a volume ratio of hydrogen peroxide and sulfuric acid (3:7) for 15 min, then thoroughly rinsed with DI water, and dried in an oven at 75 °C for 5 h. ZnCo_2_O_4_-Zn(OH)_2_ microspheres were prepared using a facile hydrothermal approach on carbon cloth. A solution consisting of 0.75 mmol of Zn(NO_3_)_2_·6H_2_O, 1.5 mmol of Co(NO_3_)_2_·6H_2_O, 1.5 mM of NH_4_F, and 3.75 mM of urea was prepared in 30 mL of DI water and stirred magnetically for 20 min. Subsequently, the carbon cloth, having undergone prior preparation, was immersed in this homogenous mixture and maintained at 120 °C for 5 h. Once the temperature returned to ambient, the ZnCo_2_O_4_-Zn(OH)_2_ microspheres were washed with DI water and ethanol and desiccated at 75 °C for the entire night. The specimen was calcinated at varying temperatures for 2 h under atmospheric pressure.

### 3.3. Characterization

Various analytical techniques thoroughly examined the microstructures and elemental composition of the as-synthesized ZnCo_2_O_4_-Zn(OH)_2_ microspheres. Field-emission scanning electron microscopy (FESEM) was performed utilizing a Hitachi S-4800 instrument from Japan. Field emission transmission electron microscopy (FETEM) utilized a JEOL-2100F instrument manufactured in Japan, outfitted with energy-dispersive X-ray spectroscopy (EDS) to analyze the primary components. X-ray diffraction (XRD) analysis was conducted utilizing a Bruker D2 instrument in the United States to examine the crystal structures of the fabricated substrates. The elemental chemical compositions of the ZnCo_2_O_4_-Zn(OH)_2_ microspheres were analyzed through X-ray photoelectron spectroscopy (XPS) utilizing a ULVAC-PHI PHI 5000 VersaProbe instrument manufactured in Japan.

### 3.4. Photocatalytic Measurement

The efficiency of photocatalytic materials was assessed through the disintegration of a methyl violet solution (0.01 mM) and tetracycline (0.1 mM) without altering the pH levels. During the standard photocatalytic activity, a UVC lamp emitting 253.7 nm with a power of 10 W from Philips in Amsterdam served as the illumination source. Changes in the distinctive absorption bands of the methyl violet and tetracycline solutions were monitored via a UV-vis spectrophotometer (Hitachi U-2900, Tokyo, Japan). When exposed to UVC lamp illumination, these photocatalysts’ effectiveness was quantified by the ratio C/C_0_, where C_0_ represents the solutions’ initial concentrations, and C denotes their concentrations at specific instances.

## 4. Conclusions

ZnCo_2_O_4_-Zn(OH)_2_ microspheres were effectively created on a carbon cloth using a facile hydrothermal method. The surface features of these microspheres and their ability to break down methyl violet were enhanced by adjusting the thermal annealing temperatures. This temperature adjustment was key in modifying the porosity of the ZnCo_2_O_4_-Zn(OH)_2_ microspheres, improving their photocatalytic abilities. The thermally annealed ZnCo_2_O_4_-Zn(OH)_2_ microspheres showed increased specific surface area and light absorption compared to untreated ones, making them highly effective UVC-light-exposed photocatalysts. Further studies confirmed the microspheres’ superior performance as reusable photocatalysts for degrading MV and TC. Trapping experiments identified superoxide and hydroxyl radicals as the major reactive species in the photodegradation process involving ZnCo_2_O_4_-Zn(OH)_2_ microspheres. ZnCo_2_O_4_-Zn(OH)_2_ microspheres offer a cost-effective and efficient photocatalytic solution with potential applications across various disciplines due to their simplicity, high efficiency, and reusability.

## Data Availability

No new data were created or analyzed in this study. Data sharing is not applicable to this article.
